# Age-Related Hearing Loss in Mn-SOD Heterozygous Knockout Mice

**DOI:** 10.1155/2013/325702

**Published:** 2013-06-27

**Authors:** Makoto Kinoshita, Takashi Sakamoto, Akinori Kashio, Takahiko Shimizu, Tatsuya Yamasoba

**Affiliations:** ^1^Department of Otolaryngology and Head and Neck Surgery, University of Tokyo, Hongo 7-3-1, Bunkyo-ku, Tokyo 113-8665, Japan; ^2^Department of Advanced Aging Medicine, Chiba University Graduate School of Medicine, 1-8-1 Inohana, Chuo-ku, Chiba 260-8670, Japan

## Abstract

Age-related hearing loss (AHL) reduces the quality of life for many elderly individuals. Manganese superoxide dismutase (Mn-SOD), one of the antioxidant enzymes acting within the mitochondria, plays a crucial role in scavenging reactive oxygen species (ROS). To determine whether reduction in Mn-SOD accelerates AHL, we evaluated auditory function in Mn-SOD heterozygous knockout (HET) mice and their littermate wild-type (WT) C57BL/6 mice by means of auditory brainstem response (ABR). Mean ABR thresholds were significantly increased at 16 months when compared to those at 4 months in both WT and HET mice, but they did not significantly differ between them at either age. The extent of hair cell loss, spiral ganglion cell density, and thickness of the stria vascularis also did not differ between WT and HET mice at either age. At 16 months, immunoreactivity of 8-hydroxydeoxyguanosine was significantly greater in the SGC and SV in HET mice compared to WT mice, but that of 4-hydroxynonenal did not differ between them. These findings suggest that, although decrease of Mn-SOD by half may increase oxidative stress in the cochlea to some extent, it may not be sufficient to accelerate age-related cochlear damage under physiological aging process.

## 1. Introduction

Age-related hearing loss (AHL), also referred to as presbycusis, is the most common cause of hearing loss in aged people. It occurs in 25–45% of people aged 65 years or older; the prevalence rises with age, ranging from 40% to 66% in people older than 75 years and more than 80% in people older than 85 years [1]. The number of people suffering from this disorder is dramatically growing as the population of older people increases in developed countries such as the USA and Japan. AHL is defined as progressive, bilateral, and symmetrical hearing impairment. It generally begins at high-frequency region and progresses toward the lower-frequency region. AHL is often associated with difficulty in speech discrimination and sound detection and localization. Ultimately, it can affect the cognitive, emotional, and social function. The pathophysiology of AHL therefore needs to be clarified to develop new therapeutic modalities.

AHL is histologically characterized by structural changes in inner ear, such as degeneration of sensory cells, auditory neurons, and cells of the stria vascularis (SV) [2–8]. Nevertheless, AHL does not occur uniformly in humans, and the participation of multiple pathological processes has been assumed. One of the causes of the age-related cochlear degeneration has been attributed to the accumulated effects of numerous insults, including exposure to intense noise and ototoxic drugs. However, age-related cochlear degeneration can occur in the absence of such insults. Hellstrom and Schmiedt [9] reported that mammals reared in quiet environments without exposure to ototoxins showed progressive hearing loss with aging, suggesting the role of genetic and intrinsic factors in addition to environmental factors.

Accumulating evidence has shown that the damage of cochlear hair cell (HC) induced by intense noise and exposure to ototoxic agents such as aminoglycosides and cisplatin is mediated through the generation of reactive oxygen species (ROS) [10–14]. The free radical theory of aging has obtained consensus also in terms of AHL by several lines of evidence [15–17]. Mitochondria are considered to play a key role in aging and AHL as a main source of ROS, and the bulk of mitochondrial ROS is generated at the electron transport chain [18, 19]. A small amount of the electrons leaking from the electron transport chain causes one-electron reduction of oxygen and produces superoxide anion (O_2_^−^) which is a short-lived free radical [20, 21]. O_2_^−^ is dismutated into hydrogen peroxide (H_2_O_2_). In the situation of respiratory chain dysfunction due to mutations, increase of O_2_^−^ production can lead to the accumulation of H_2_O_2_ and other ROS. Once produced, ROS react with macromolecules such as lipid, DNA, and proteins. In particular, mitochondrial DNA (mtDNA) is vulnerable [22–24] to oxidative stress because it is located in the site of oxidative phosphorylation. Damage of mtDNA causes further dysfunction of mitochondria and augments oxidative stress. In the cochlea, cells of the SV [25], spiral ganglion cells (SGCs) [26, 27], and HCs [28] all contain numerous mitochondria and are considered to be susceptible to ROS-induced damage. Moreover, mtDNA damage has been reported to induce apoptosis of the important cochlear structures such as the SGCs [29]. These suggest that mtDNA damage induced by excess generation of ROS is one of the leading causes of AHL [29].

There are several enzymatic antioxidant defense systems, including copper/zinc superoxide dismutase (Cu/Zn-SOD, SOD1), manganese superoxide dismutase (Mn-SOD), catalase, and peroxidase, which convert ROS to neutral and nonreactive molecules. These antioxidant enzymes are considered to be important contributors to cellular homeostasis. Nevertheless, the roles of the antioxidant enzymes in the free radical theory of aging are controversial because of the inconsistent findings. For example, mice heterozygous for Mn-SOD have reduced activity of the enzyme, increased oxidative damage, but normal life span [30]. Overexpression of antioxidant enzymes Cu/Zn-SOD does not extend lifespan in mice [31]. The median lifespan of mice heterozygous of glutathione peroxidase 4 is significantly longer than that of wild-type mice in spite of increased sensitivity to oxidative stress-induced apoptosis [32].

Several previous studies assessed the importance of antioxidant enzymes in AHL. In Cu/Zn-SOD transgenic mice, absence of Cu/Zn-SOD resulted in a very large loss of auditory neurons and HCs and an early onset of hearing loss [33–35]. Conversely, heterozygous Cu/Zn-SOD knockout mouse, which had reduced expression of the enzyme, maintained hearing and normal cochlear morphology [33]. These results suggest that even half as much Cu/Zn-SOD is sufficient to maintain cochlear function and morphology under normal physiological condition.

To date, only a few studies have been available which examined the role of Mn-SOD in AHL. Mn-SOD, one of the antioxidant enzymes located in the mitochondrial matrix, plays an important role to protect mtDNA from oxidative stress. Le and Keithley [36] evaluated the hearing function in Mn-SOD heterozygous knockout mice and found no difference in the extent of hearing loss when compared to the background strain, although they did not examine the histological and immunohistochemical findings in the cochlea. We hypothesize that histological and immunohistochemical evaluation for oxidative markers would detect subtle changes reflecting the reduction of Mn-SOD even when functional assessments fail to detect the differences between Mn-SOD heterozygous knockout mice and the background strain.

In the present study, we conducted histological evaluation and immunostaining using anti-Mn-SOD, anti-4-HNE, and 8-OHdG antibody of the cochlea, as well as functional assessment by auditory brainstem response (ABR) in Mn-SOD heterozygous knockout mice and compared them with those in the background strain C57BL/6 mice.

## 2. Materials and Methods

### 2.1. Animals

Mn-SOD lox/lox mice were generated by one of the authors (Takahiko Shimizu) at the Molecular Gerontology Laboratory in Tokyo Metropolitan Institute of Gerontology, as described previously [37]. These mice were backcrossed with C57BL/6NCrSlc mice for five or six generations. The crossbreeding of homozygous Mn-SOD lox/lox mice with the chicken actin promoter (CAG)-Cre transgenic mice [38] of a C57BL/6 background gave rise to systemic heterozygous Mn-SOD-deficient (HET) mice. These mice presented systemically only half as much Mn-SOD. As a control, their littermate wild-type (WT) C57BL/6 mice were used. All animals were kept at 22 ± 1°C under a 12-hour light/12-hour dark cycle and had free access to water and regular mouse diet (MF, Oriental Yeast Co., Tokyo, Japan).

We employed a total of 22 mice in the current study: 4-month-old WT group (*n* = 6), a 16-month-old WT group (*n* = 5), a 4-month-old HET group (*n* = 6), and a 16-month-old HET group (*n* = 5). All animals underwent ABR assessment, after which they were euthanized for evaluation of cochlear pathology and immunohistochemistry.

Experiment protocol was approved by the Institutional Review Board of the Faculty of Medicine, University of Tokyo. All the procedures were performed in accordance with the guidelines of the University Committee for the Use and Care of Animals, University of Tokyo, and the National Institutes of Health Guide for the Care and Use of Laboratory Animals.

### 2.2. Assessment of Hearing Function

Detailed protocols for ABR measurements have been described elsewhere [39]. Briefly, two examiners who were blind to the experiment measured ABRs with a tone burst stimulus (4, 8, 16, and 32 kHz) using an ABR recording system (Neuropack Σ MEB5504, Nihon Kohden, Tokyo, Japan). Mice were anesthetized with a mixture of xylazine hydrochloride (10 mg/kg, i.m.) and ketamine hydrochloride (40 mg/kg, i.m.). Needle electrodes were placed subcutaneously at the vertex (active electrode), beneath the left pinna (reference electrode), and beneath the right ear (ground). The stimulus duration was 15 ms. Responses of 1024 sweeps were averaged at each intensity level (5 dB steps) to assess threshold. Threshold was defined as the lowest intensity level at which a clear reproducible waveform was visible in the trace. ABR thresholds were measured for WT and HET mice at 4 or 16 months of age. To obtain ABR input/output (I/O) functions, the wave I peak amplitude was identified by visual inspection at each stimulus level. All data were reported as mean ± SD.

### 2.3. Histological Evaluation and Immunostaining

After ABR measurements, all animals were euthanized under deep anesthesia with xylazine hydrochloride and ketamine hydrochloride, and the cochleae were dissected out. They were immersed in 4% paraformaldehyde in 0.1 M phosphate-buffered saline (PBS) overnight at 4°C and decalcified in 10% ethylenediaminetetraacetic acid (EDTA) solution. The specimens were then dehydrated through a graded alcohol series and embedded in paraffin. The embedded tissues were cut into 5 *μ*m thick sections parallel to the modiolus, and every three sections were mounted on glass slides and deparaffinized. Every three slides containing 5 sections were stained with hematoxylin and eosin and observed under a light microscope (Nikon Eclipse E800M, 40x obj.) for evaluating HC survival rates, SGC densities, and strial thicknesses. The other slides were used for immunohistochemical evaluation. We used samples from 6 mice from 4-month-old groups and 5 mice from 16-month-old groups. All data were reported as mean ± SD.

#### 2.3.1. Hair Cell Survival Rate

HCs were counted as present if the cell body and cuticular plate looked intact. The number of remaining HCs at the lower-basal, upper-basal, and lower-middle turns was counted at least in 10 sections per animal. We calculated the inner and outer hair cell (IHC and OHC) survival rates of these three turns in each animal by using the following formulae: IHC survival rate% = 100 × [(the number of present IHCs of examined specimens)/the number of examined specimens]; OHC survival rate% = 100 × [(the number of present OHCs of examined specimens)/the number of examined specimens/3].

#### 2.3.2. Spiral Ganglion Cell Density

An unbiased investigator inspected the collection of mid-modiolar sections generated for each cochlea and selected the slides that had high-quality sections. From those selected, three sections from each cochlea were randomly chosen for counting. The number of SGCs and the area of Rosenthal's canal of the basal turns were measured in digital photomicrographs (40x obj.) using Photoshop CS4 software, and SGC density (SGC number/mm^2^) was calculated, as previously reported [40]. SGCs in each profile were counted on the computers monitor. The area (mm^2^) of the Rosenthal's canal profile was measured in each photomicrograph by outlining the margin of bony canal with a calibrated computer mouse. The area of the outline was calculated using Photoshop CS4 software. The density of SGC was calculated for each profile of the ganglion.

#### 2.3.3. Stria Vascularis Thickness

The thicknesses of the SV in radial sections of the basal turns were measured in digital photomicrographs (40x obj.) using Photoshop CS4 software. A line was drawn from the strial margin to the spiral ligament junction half way between the attachment of Reissner's membrane and the spiral prominence using a calibrated computer mouse, and the length of the line was calculated by the computer. Three sections from each turn in each cochlea were measured.

#### 2.3.4. Immunohistochemistry

The cochleae were fixed in PBS-buffered 4% paraformaldehyde for 24 hrs and decalcified with 10% EDTA solution (pH 7.0). After embedding in paraffin, 5 *μ*m sections were cut and mounted on silane-coated slides. Deparaffinized sections were autoclaved in citrate-buffered saline (PH 4.0) for 20 minutes for antigen retrieval. Immunohistochemistry was performed using either of the following antibodies: anti-Mn-SOD antibody (rabbit monoclonal antibody, Epitomics Inc., San Francisco CA, USA; 1 : 100 dilution), anti-8-OHdG antibody (goat polyclonal antibody, Alpha Diagnostic International Inc., San Antonio, TX, USA; 1 : 100 dilution), and anti-4-HNE antibody (rabbit polyclonal antibody, Alpha Diagnostic International Inc., San Antonio, TX, USA; 1 : 100 dilution). Immunoreaction was detected using the following secondary antibody systems: Histofine Simple Stain MAX-PO (G) (Nichirei Corp., Tokyo, Japan). Ten randomly selected high-power fields (×400) from three section prepared from each mouse were examined under light microscope. The labeling index of each antibody was obtained by a modified Photoshop-based image analysis as described previously [41].

### 2.4. Statistical Analysis

SigmaStat statistical software was used and all data were expressed as mean ± SD. ABR thresholds, HC survival rates, SGC densities, and SV thicknesses were compared among groups by two-way analysis of variance (ANOVA), and then pairwise comparisons were performed by using Scheffe's test.

## 3. Results

### 3.1. Systemic Findings

Generally, WT and HET mice looked similar at 4 months and 16 months of age. The mean body weights of 4-month-old WT and HET mice and 16-month-old WT and HET mice were 24.7 ± 1.03 g (range 23 to 26 g), 24.5 ± 1.52 g (range 23 to 27 g), 30.6 ± 1.52 g (range 29 to 33 g), and 31.4 ± 1.67 g (range 30 to 34 g), respectively. The body weights did not significantly differ between HET and WT mice at either age.

### 3.2. Hearing Function

**Figure 1:**
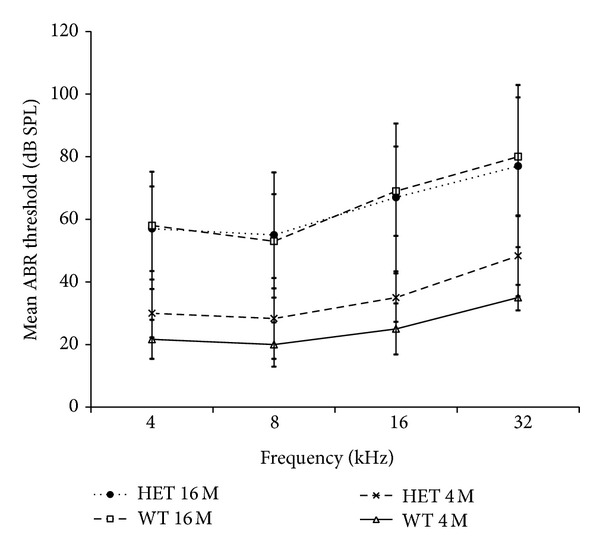
ABR thresholds (mean ± SD) of HET and WT mice at 4 and 16 months of age.

HET and WT mice showed nearly normal ABR thresholds at 4, 8, and 16 kHz and only slightly increased thresholds at 32 kHz at 4 months of age (Figure 1). At 16 months, ABR thresholds were significantly increased at all frequencies tested in both HET and WT mice, but they did not significantly differ between HET and WT mice at either age at any frequency tested.

**Figure 2:**
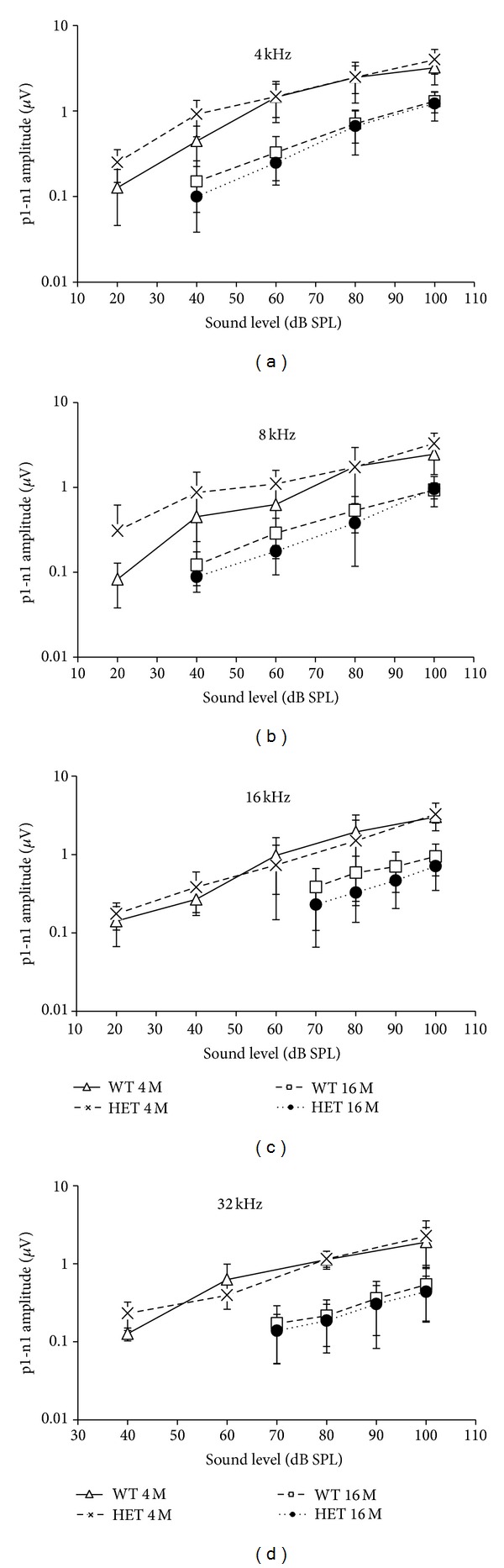
ABR wave I amplitude I/O functions (mean ± SD) at 4, 8, 16, and 32 kHz in HET and WT mice at 4 and 16 months of age.

We also employed ABR wave I amplitude I/O functions to assess the gross activity of the mouse auditory nerve. As shown in Figures 2(a)–2(d), the slopes of the I/O functions were similar between WT and HET mice at all frequencies tested at either 4 or 16 months of age. The maximum wave I amplitudes were reduced significantly at all frequencies in 16-month-old WT and HET mice compared to 4-month-old WT and HET mice, but they did not significantly differ between HET and WT mice at either age.

### 3.3. Hair Cell Survival Rate

**Figure 3:**
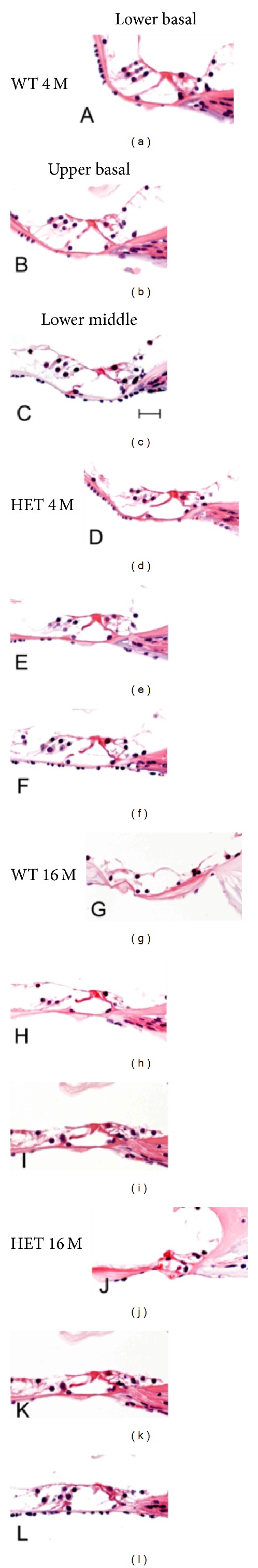
Representative photomicrographs of the organ of Corti in the lower-basal, upper-basal, and lower-middle turns from 4- and 16-month-old WT and HET mice. Scale bar: 25 *μ*m.

**Figure 4:**
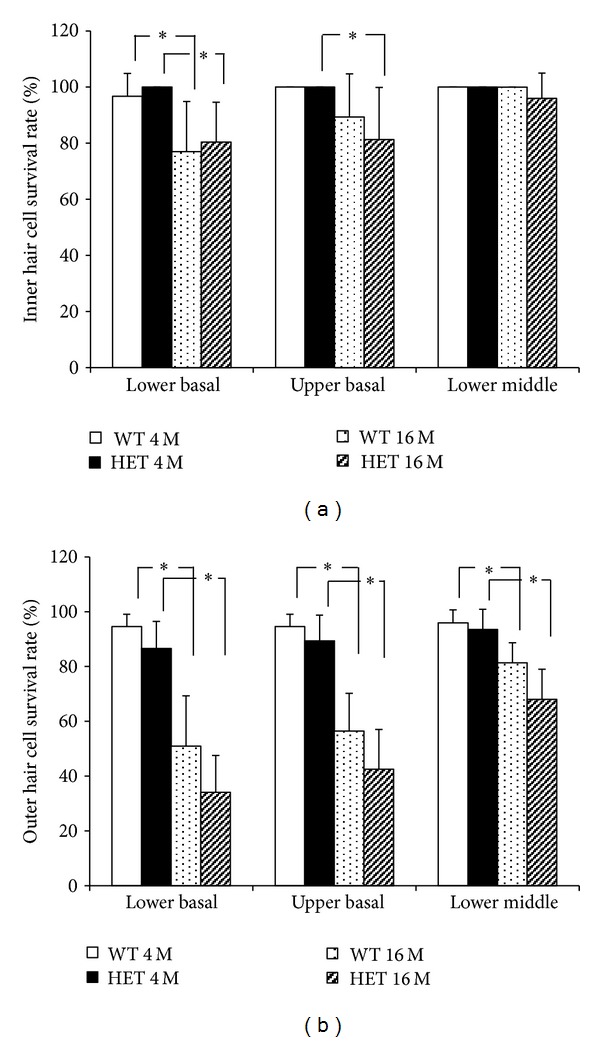
The survival rates (mean ± SD) of the inner (a) and outer (b) hair cells in the lower-basal, upper-basal, and lower-middle turns from 4- and 16-month-old WT and HET mice from each experimental group. *: *P* < 0.05.

Both 16-month-old WT and HET mice displayed loss of IHCs in the basal turn, more significantly in the lower-basal turn (Figures 3(g), 3(j)), whereas 4-month-old WT and HET mice displayed no or only a little loss of IHCs in all turns (Figures 3(a)–3(f), 3(h), 3(i), 3(k), and 3(l)). The IHC survival rates in 4-month-old WT and HET mice and 16-month-old WT and HET mice were 96.7 ± 8.16%, 100 ± 0%, 77.0 ± 17.9%, and 80.4 ± 14.2% in the lower-basal turn, respectively; 100 ± 0%, 100 ± 0%, 89.3 ± 15.4%, and 81.3 ± 13.2% in the upper-basal turn, respectively; and 100 ± 0%, 100 ± 0%, 100 ± 0%, and 96 ± 8.9% in the lower-middle turn, respectively. The IHC survival rates were significantly reduced at the lower-basal and upper-basal turns in 16-month-old HET mice when compared to 4-month-old HET mice. They were also significantly reduced at the lower-basal turn in 16-month-old WT mice compared to 4-month-old WT mice. However, the extent of IHC loss did not significantly differ between HET and WT mice at either age in any of the turns (Figure 4(a)). 

Similar trends were also observed in the extent of OHC loss. Both 16-month-old WT and HET mice displayed severe loss of OHCs (Figures 3(g)–3(l)), whereas 4-month-old WT and HET mice displayed no or only a little loss of OHCs (Figures 3(a)–3(f)). The OHC survival rates in 4-month-old WT and HET mice and 16-month-old WT and HET mice were 94.5 ± 4.5%, 86.6 ± 9.9%, 51.0 ± 18.3%, and 34.2 ± 13.4% in the lower-basal turn, respectively; 94.5 ± 4.5%, 89.4 ± 9.4%, 56.4 ± 13.8%, and 42.6 ± 14.4% in the upper-basal turn, respectively; and 95.9 ± 4.8%, 93.5 ± 7.4%, 81.3 ± 7.3%, and 68.0 ± 11.0% in the lower-middle turn, respectively. In either HET or WT mice, the OHC survival rates were significantly reduced in all turns at 16 months when compared to those at 4 months. However, they were not significantly different between HET and WT mice at either age in all turns (Figure 4(b)).

### 3.4. Spiral Ganglion Cell Density

**Figure 5:**
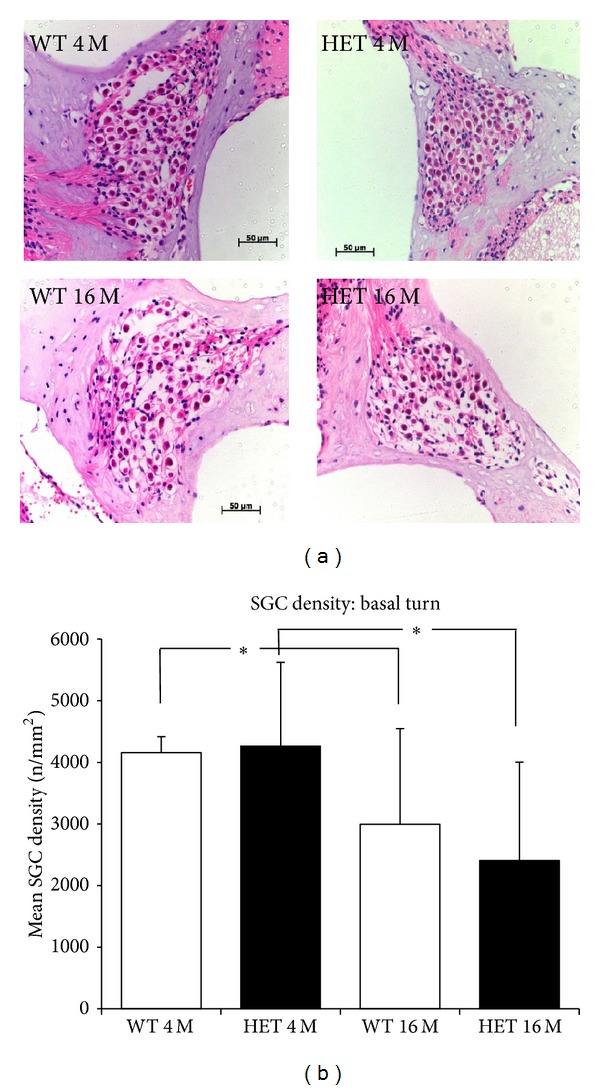
(a) Representative photomicrographs of Rosenthal's canal in the basal turn from 4- and 16-month-old WT and HET mice. Scale bar: 50 *μ*m. (b) SGC densities (mean ± SD) in the modiolar sections from the basal turn in 4- and 16-month-old WT and HET mice. *: *P* < 0.05.

At 4 months of age, the mean SGC densities of the basal turn were 4,263 ± 1,360/mm^2^ and 4,157 ± 259/mm^2^, and those of the middle turn were 5,431 ± 117/mm^2^ and 5269 ± 417/mm^2^ in the HET and WT mice, respectively. At 16 months, the mean SGC densities of the basal turn were 2,407 ± 1,595/mm^2^, and 2,993 ± 1,554/mm^2^, and those of the middle turn were 5,269 ± 417/mm^2^ and 4,134 ± 583/mm^2^ in the HET and WT mice, respectively. In either HET or WT mice, the SGC densities were significantly lower in both the basal and apical turns at 16 months compared to those at 4 months; however, they were not significantly different between HET and WT mice at either age (Figures 5(a) and 5(b)).

### 3.5. Stria Vascularis Thickness

**Figure 6:**
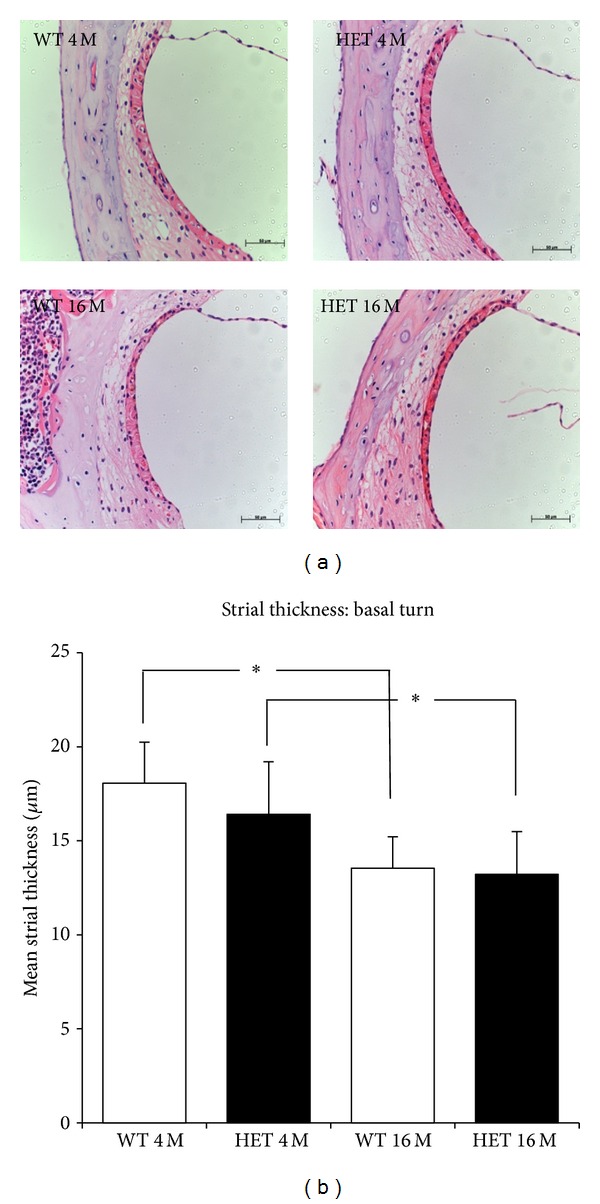
(a) Representative photomicrographs of SV in the basal turn from 4- and 16-month-old WT and HET mice. Scale bar: 50 *μ*m. (b) Strial thickness (mean ± SD) in the modiolar sections from the basal turn in 4- and 16-month-old WT and HET mice. *: *P* < 0.05.

At 4 months of age, the strial thickness in the basal turn was 16.4 ± 2.8 *μ*m in the HET mice and 18.1 ± 2.1 *μ*m in the WT mice, and that in the middle turn was 18.5 ± 3.4 *μ*m in the HET mice and 18.8 ± 1.6 *μ*m in the WT mice. At 16 months, the strial thickness in the basal turn was 13.2 ± 2.3 *μ*m in the HET mice and 13.5 ± 1.7 *μ*m in the WT mice, and that in the middle turn was 15.2 ± 1.8 *μ*m in the HET mice and 17.2 ± 3.1 *μ*m in the WT mice. While the strial thickness was significantly (*P* < 0.05) decreased from 4 months to 16 months in both HET and WT mice, it was not significantly different between HET and WT mice at either age (Figures 6(a) and 6(b)).

### 3.6. Expression of Mn-SOD

**Figure 7:**
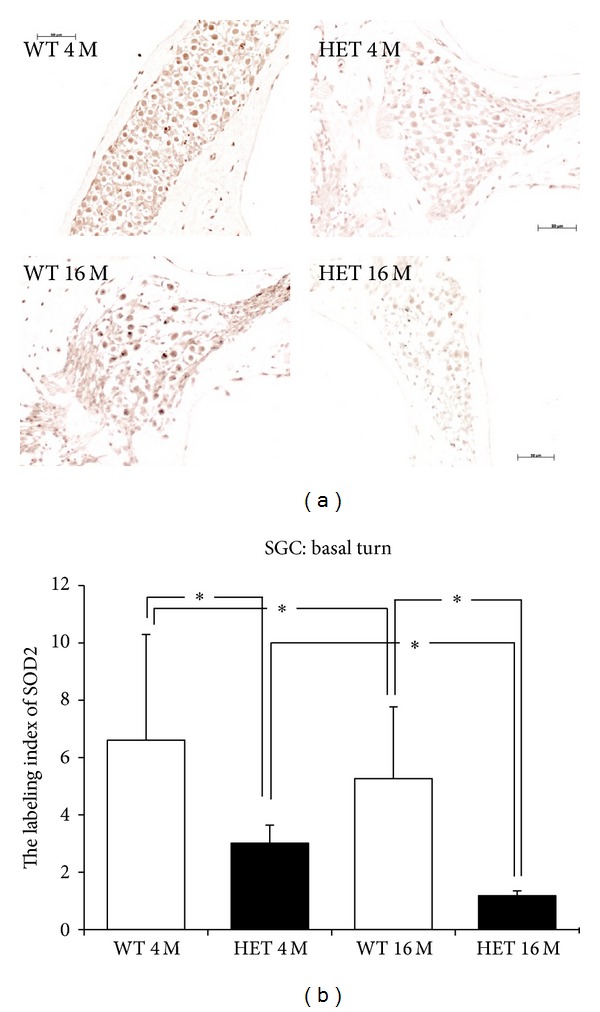
(a) Representative photomicrographs of immunostaining with anti-Mn-SOD antibody of the SGC in the basal turn from 4- and 16-month-old WT and HET mice. Scale bar: 50 *μ*m. (b) SGC labeling indices (mean ± SD) of Mn-SOD in the basal turn in 4- and 16-month-old WT and HET mice. *: *P* < 0.05.

In anti-Mn-SOD immunostaining, immunopositive cells were more abundant in the SGC in the basal turn in WT mice compared to HET mice at both ages (Figure 7(a)). The labeling indices of Mn-SOD at 4 months were 6.61 ± 3.69 in WT mice and 3.01 ± 0.64 in HET mice, and those at 16 months were 5.27 ± 2.50 in WT mice and 1.18 ± 0.17 in HET mice (Figure 7(b)). In HET mice, the labeling indices of Mn-SOD were 45.6% and 22.5% of those in WT mice at 4 and 16 months of age, respectively, with statistically significant differences between them at either age (*P* < 0.05). From 4 months to 16 months of age, the labeling indices of Mn-SOD showed 20.3% decrease in WT mice and 60.8% decrease in HET mice; the labeling indices of Mn-SOD differed significantly (*P* < 0.05) between 4 and 16 months in both WT and HET mice.

**Figure 8:**
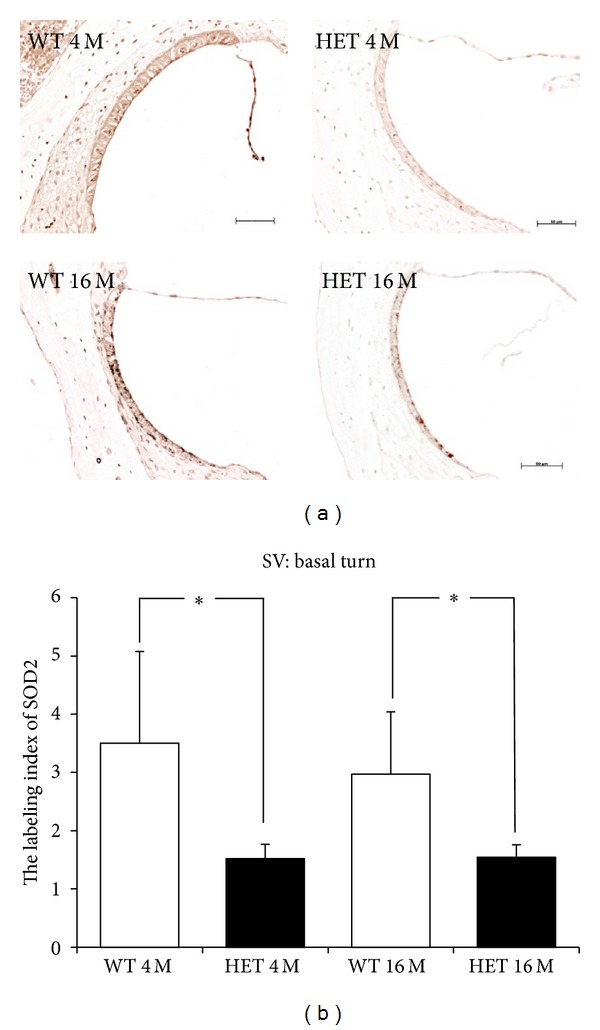
(a) Representative photomicrographs of immunostaining with anti-Mn-SOD antibody of the SV in the basal turn from 4- and 16-month-old WT and HET mice. Scale bar: 50 *μ*m. (b) SV labeling indices (mean ± SD) of Mn-SOD in the basal turn in 4- and 16-month-old WT and HET mice. *: *P* < 0.05.

In the SV in the basal turn, the immunoreactivities of Mn-SOD were markedly greater in WT mice compared to HET mice at both ages (Figure 8(a)). The labeling indices of Mn-SOD at 4 months were 3.50 ± 1.58 in WT mice and 1.52 ± 0.24 in HET mice, and those at 16 months were 2.97 ± 1.27 in WT mice and 1.54 ± 0.22 in HET mice (Figure 8(b)). In HET mice, the labeling indices of Mn-SOD were 43.4% and 51.9% of those in WT mice at 4 and 16 months of age, respectively, with statistically significant differences between them at either age (*P* < 0.05). Different from the SGC, the labeling indices of Mn-SOD in SV did not significantly change from 4 months to 16 months in either WT or HET mice.

### 3.7. Expression of 8-OHdG

**Figure 9:**
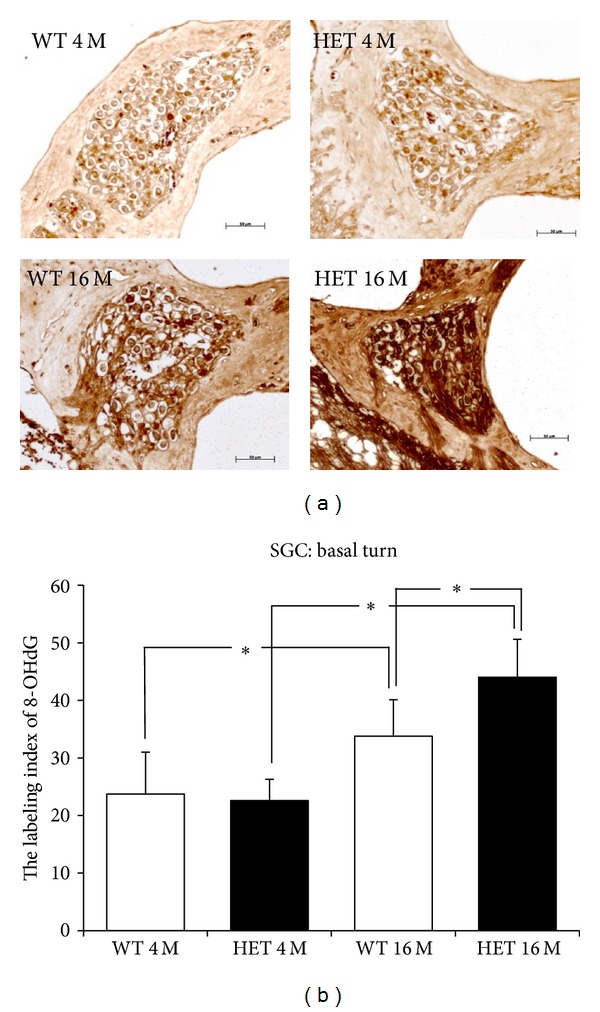
(a) Representative photomicrographs of immunostaining with anti-8-OHdG antibody of the SGC in the basal turn from 4- and 16-month-old WT and HET mice. Scale bar: 50 *μ*m. (b) SGC labeling indices (mean ± SD) of 8-OHdG for the basal turn in 4- and 16-month-old WT and HET mice. *: *P* < 0.05.

In anti-8-OHdG immunostaining, in the SGC in the basal turn, the immunopositive cells were more abundant at 16 months compared to 4 months in both mice (Figure 9(a)). The labeling indices of 8-OHdG in WT and HET mice at 4 months were 23.7 ± 7.3 and 22.6 ± 3.7, respectively, and those at 16 months were 33.8 ± 6.3 and 44.0 ± 6.6, respectively (Figure 9(b)). The labeling indices of 8-OHdG at 16 months were significantly (*P* < 0.05) greater compared to those at 4 months in both mice. Also, the labeling indices of 8-OHdG showed a significant difference (*P* < 0.05) between HET and WT mice at 16 months of age. 

**Figure 10:**
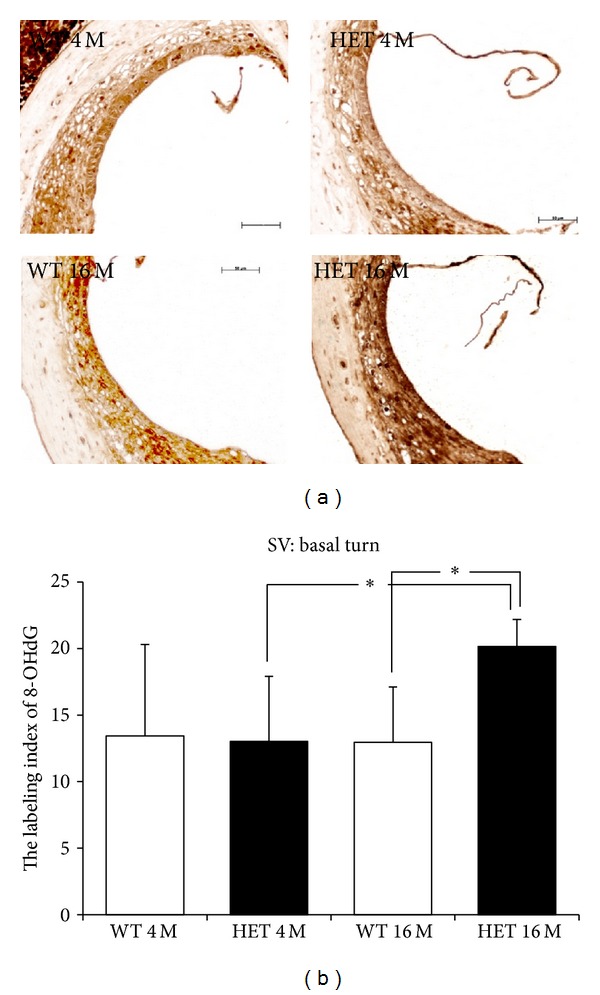
(a) Representative photomicrographs of immunostaining with anti-8-OHdG antibody of the SV in the basal turn from 4- and 16-month-old WT and HET mice. Scale bar: 50 *μ*m. (b) SV labeling indices (mean ± SD) of 8-OHdG for the basal turn in 4- and 16-month-old WT and HET mice. *: *P* < 0.05.

In the SV in the basal turn, the immunoreactivity was the strongest in 16-month-old HET mice, while the immunoreactivity was almost the same among WT mice at both 4 and 16 months and HET mice at 4 months (Figure 10(a)). In the SV in the basal turn, the labeling indices of 8-OHdG of WT and HET mice were 13.4 ± 6.9 and 13.0 ± 4.9 at 4 months, respectively, and were 13.0 ± 4.2 and 20.2 ± 2.0 at 16 months, respectively (Figure 10(b)). The labeling indices of 8-OHdG in HET mice at 16 months were significantly (*P* < 0.05) greater compared to those at 4 months. Also, the labeling indices of 8-OHdG were significantly different (*P* < 0.05) between HET and WT mice at 16 months.

### 3.8. Expression of 4-HNE

**Figure 11:**
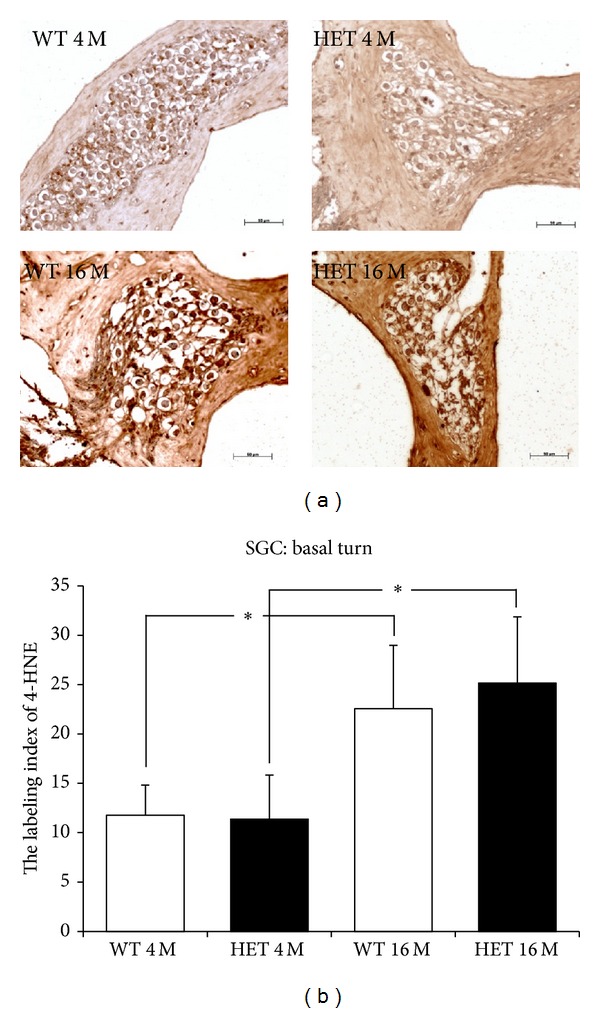
(a) Representative photomicrographs of immunostaining with anti-4-HNE antibody of the SGC in the basal turn from 4- and 16-month-old WT and HET mice. Scale bar: 50 *μ*m. (b) SGC labeling indices of 4-HNE for the basal turn in 4- and 16-month-old WT and HET mice. *: *P* < 0.05.

In anti-4-HNE immunostaining, in the SGC in the basal turn, immunopositive cells were more abundant at 16 months of age compared to 4 months of age in both WT and HET mice (Figure 11(a)). The labeling indices of 4-HNE in WT and HET mice at 4 months were 11.8 ± 3.1 and 11.4 ± 4.5, respectively, and those at 16 months were 22.6 ± 6.4 and 25.2 ± 6.7, respectively (Figure 11(b)). The labeling indices of 4-HNE at 16 months in both WT and HET mice were significantly (*P* < 0.05) greater than those at 4 months, but there were no significant differences between HET and WT mice at either age. 

**Figure 12:**
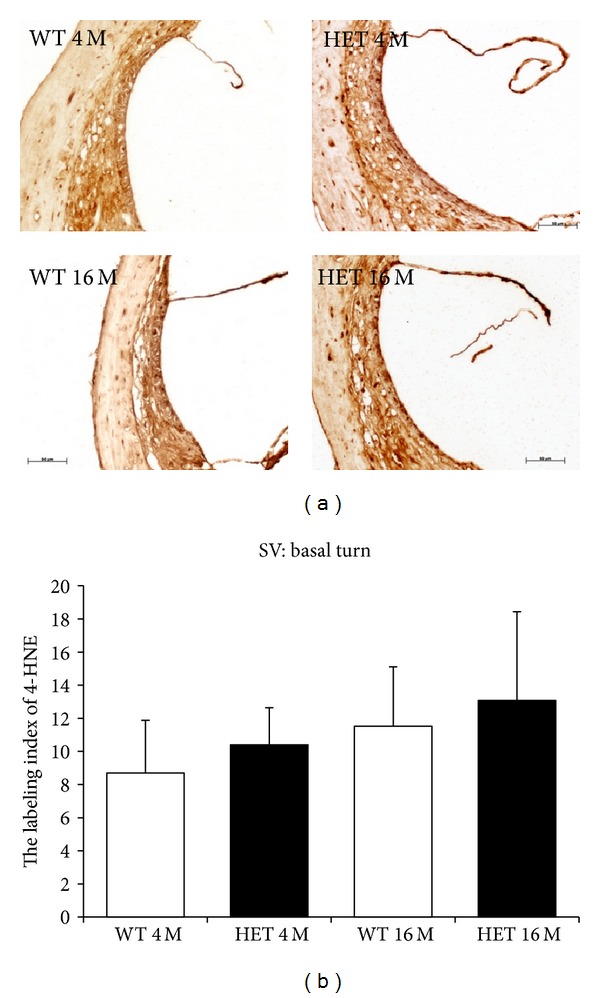
(a) Representative photomicrographs of immunostaining with anti-4-HNE antibody of the SV in the basal turn from 4- and 16-month-old WT and HET mice. Scale bar: 50 *μ*m. (b) SV labeling indices of 4-HNE in the basal turn in 4- and 16-month-old WT and HET mice.

In the SV in the basal turn, the labeling indices of 4-HNE in WT and HET at 4 months were 8.70 ± 3.2 and 10.4 ± 2.2, respectively, and those at 16 months were 11.5 ± 3.6 and 13.1 ± 5.4, respectively (Figure 12(b)). The labeling indices of 4-HNE tended to be greater in HET mice than in WT mice at either age and tended to increase with age in both HET and WT mice, but they were not significantly different between WT and HET mice at either age or between 4 and 16 months of age in either of the mice.

## 4. Discussion

In the current study, HET mice did not show acceleration in ABR threshold shifts with aging when compared to WT mice. This physiological finding was confirmed by histological analysis, which revealed no significant differences in HC survival rates, SGC density, and SV thickness between HET and WT mice at either 4 or 16 months of age. The HET mice had reduced Mn-SOD activity (~50%) in the SGC and SV at either age when compared to WT mice. The expression of 8-OHdG, a marker of DNA oxidation, was increased in the SGC with aging in both WT and HET mice, and the expression of 8-OHdG in the SGC and SV at 16 months was significantly greater in HET mice compared to WT mice. The expression of 4-HNE, a marker of lipid peroxidation, was increased in the SGC with aging and tended to increase in the SV in both HET and WT, although it was not significantly different between HET and WT mice. These findings suggest that half reduction of Mn-SOD may accelerate oxidative stress, predominantly to DNA, to some extent, but may not be sufficient to increase the damage to the cochlear tissues under normal aging process. Since both WT and HET mice carry a specific mutation in the cadherin 23 gene, it is possible that the effect of Mn-SOD might be masked by C57 genetic pathology. However, we enrolled wild-type C57BL/6 littermates as a control, which we consider made the influence of the C57 background minimal. 

It has been reported that mice lacking Cu/Zn-SOD exhibited 30% decrease in life expectancy [42], whereas overexpression of Cu/Zn-SOD and catalase extended lifespan in *Drosophila* [43]. Further, small synthetic mimetics of SOD/catalase increased lifespan in *C. elegans* [44]. Collectively, these results imply that interplay between ROS and protective responses by antioxidant enzymes is an important factor in determining aging and lifespan. Nevertheless, the role of these antioxidant enzymes in aging is still controversial. *Sod2+/− *mice have been reported to have reduced Mn-SOD activity (~50%) in all tissues throughout life, increased oxidative damage as demonstrated by elevation of 8-OHdG in all tissues (significantly higher compared with WT mice), and increase in tumor incidence. However, the lifespans of *Sod2+/− *mice were identical to those of WT mice and biomarkers of aging, such as cataract formation, immune response, and formation of glycoxidation products carboxymethyl lysine and pentosidine in skin collagen changed with age to the same extent in both WT and *Sod2+/− *mice [30], indicating that life-long reduction of Mn-SOD activity leads to increased levels of oxidative damage to DNA and increased cancer incidence but does not appear to affect aging. Overexpression of antioxidant enzymes in mice, such as Cu/Zn-SOD or catalase, did not extend lifespan [31, 45]. The median lifespan of mice heterozygous of glutathione peroxidase 4 was significantly longer than that of wild-type mice, even though they showed increased sensitivity to oxidative stress-induced apoptosis [32]. These results pose a question in terms of the importance of antioxidant enzymes in preventing aging process. 

As of age-related degeneration in the cochlea, it has been reported that overexpression of Cu/Zn-SOD did not prevent or slow AHL, whereas Cu/Zn-SOD KO mice exhibited acceleration of AHL due to massive loss of HCs and auditory neurons in an earlier onset [34]. Interestingly, half-expressed Cu/Zn-SOD did not accelerate AHL in mice [33]. Similarly, Le and Keithley [36] reported that Mn-SOD heterozygous transgenic mice showed no deterioration in the extent of ABR threshold shifts compared to the background strain but did not address the histological findings. Although it is ideal to investigate Mn-SOD homozygous knockout mice to assess the importance of Mn-SOD in AHL, systemic Mn-SOD-deficient mice are known to die at an early stage after birth. Because of this reason, we are forced to use Mn-SOD heterozygous knockout mice, but we examined not only their auditory function but also cochlear histology and immunohistology. The present study demonstrated no significant difference in ABR thresholds between HET and WT mice at either 4 or 16 months of age, which is consistent with the report by Le and Keithley [36]. We also found no significant differences in SGC densities or SV thickness between HET and WT mice at either 4 or 16 months, supporting that reduction of Mn-SOD by half did not accelerate age-related damage in the cochlea. 

In the present study, the labeling indices of Mn-SOD in the SGC and SV in HET mice were reduced to be ~50% of those of WT mice at either 4 or 16 months of age. The expression of Mn-SOD was declined with aging in the SGC in both WT and HET mice, whereas it was unchanged with aging in the SV. These results are in harmony with the report by Jiang et al. [46] that immunoreactivity of Mn-SOD was decreased with aging in the SGC but not in the SV in CBA/J mice. These suggest that age-related decline of Mn-SOD expression differs among tissues even in the same organ, namely, the cochlea. 

In the SGC, the expression of 8-OHdG was significantly increased with aging in both WT and HET mice and was significantly greater in HET mice at 16 months compared to WT mice. On the other hand, in the SV, the expression of 8-OHdG was significantly increased with aging only in HET mice, being significantly greater at 16 months in HET mice compared to WT mice. These findings suggest that, under normal condition, 8-OHdG accumulates steadily in the SGC but not significantly in the SV. This appears reasonable, considering that Mn-SOD is decreased with aging in the SGC but not in the SV. Under pathological situation that Mn-SOD is decreased by half, 8-OHdG may accumulate with aging more significantly in the SGC and even in the SV. 

The expression of 4-HNE was increased with aging in the SGC in both WT and HET mice, and there was no significant difference in the expression level between these mice. The expression of 4-HNE did not significantly differ in the SV between WT and HET mice at any age, although it was slightly greater in HET mice than in WT mice at either 4 or 16 months of age and slightly increased with aging in both HET and WT mice. These findings suggest that, under normal condition, 4-HNE accumulates with aging in the SGC but not significantly so in the SV. It is unclear why reduction of Mn-SOD by half increased the expression of 8-OHdG but not 4-HNE in the SGC with aging. Downregulation of Mn-SOD might contribute to the augmentation of oxidative DNA stress more significantly than lipid peroxidation in the cochlear tissues. 

Although we did not find significant differences of ABR threshold shift or the extent of degeneration of the SGC and SV between HET and WT mice under normal physiological condition, it is possible that reduction of Mn-SOD by half accelerates age-related cochlear damage under pathological condition. It has been reported that, when Mn-SOD heterozygote mice were administered hepatotoxic agent that promoted ROS generation, liver damage became prominent [47], although liver-specific Mn-SOD homozygous knockout mice presented no obvious morphological or biochemical damages under normal environmental stress [37]. This result implies that, even if tissue damage is not evident under normal condition, it can become prominent under the burden of oxidative stress. It is well known that inner ear damage by intense noise and ototoxic drugs is mediated through excessive generation of ROS [48]. To verify the hypothesis, functional and morphological assessment should be carried out in Mn-SOD homozygous knockout mice under pathological oxidative stress. We are now conducting an experiment to investigate whether HET mice will exhibit acceleration of AHL compared to WT mice when they are raised in noisy environment.

## 5. Conclusion

The ABR thresholds were significantly increased from 4 months to 16 months in both WT and HET mice, but they did not significantly differ between WT and HET mice at either age. The HC survival rates, SGC density, and strial thickness did not differ between WT and HET mice at either age. At 16 months, immunoreactivity of 8-hydroxydeoxyguanosine was significantly greater in the SGC and SV in HET mice compared to WT mice, but that of 4-hydroxynonenal did not differ between them. These findings suggest that, although decrease of Mn-SOD by half may increase oxidative stress in the cochlea to some extent, it may not be sufficient to accelerate age-related cochlear damage under physiological aging process. Further study is needed to examine if reduction of Mn-SOD may accelerate AHL under pathological condition such as in a noisy environment.
